# Extensive Transcriptome Changes Underlying the Flower Color Intensity Variation in *Paeonia ostii*

**DOI:** 10.3389/fpls.2015.01205

**Published:** 2016-01-06

**Authors:** Lexuan Gao, Hongxing Yang, Hongfeng Liu, Ji Yang, Yonghong Hu

**Affiliations:** ^1^Shanghai Key Laboratory of Plant Functional Genomics and Resources, Shanghai Chenshan Plant Science Research Center, Chinese Academy of SciencesShanghai, China; ^2^School of Landscape Architecture, Beijing Forestry UniversityBeijing, China; ^3^Center for Evolutionary Biology and Institute of Biodiversity Science, Fudan UniversityShanghai, China

**Keywords:** *Paeonia ostii*, flower color intensity, anthocyanin pigmentation, transcriptome changes, anthocyanin repressor

## Abstract

Tree peonies are a group of traditional ornamental plants, especially in East Asia, with *Paeonia ostii* as one of the most important ancestral species. *P. ostii* has flowers with varying colors, ranging from nearly white, light pink to deep pink. However, few studies have been done to unravel the molecular mechanisms underlying the flower color intensity variation in plants. Based on comparative analyses of the pigment composition and transcriptomes of *P. ostii* with different flower color intensities, we found that the anthocyanin concentration was significantly correlated with the flower color intensity in *P. ostii*. Transcriptome analysis by RNA-Sequencing revealed 7187 genes that were differentially expressed between flowers with different color intensities. Functional enrichment analysis of differentially expressed genes revealed multiple pathways possibly responsible for color intensity variation in *P. ostii*, including flavonoid biosynthesis, fatty acid oxidation, carbohydrate metabolism, and hormone-mediated signaling. Particularly, while anthocyanin biosynthesis genes showing positive correlations between their expression and anthocyanin concentration in flowers, two transcription factors, PoMYB2 and PoSPL1, seem to negatively regulate anthocyanin accumulation by affecting the activation capacity of the MYB-bHLH-WDR complex, exhibiting an inverse relationship between their expression and anthocyanin accumulation. Our results showed that, although anthocyanin biosynthesis had a direct effect on the pigmentation of the *P. ostii* flower, other metabolic and hormone-mediated signaling pathways were also contributed to the flower color intensity variation in *P. ostii*, suggesting complex coordinated changes in the transcriptional network. Differential expression of genes encoding anthocyanin repressors seems to be the major factor responsible for the intensity variation in anthocyanin pigmentation in *P. ostii*.

## Introduction

Flower color is one of the most attractive sceneries in nature. It confers flowers with diverse functions (Winkel-Shirley, 2001; Steyn et al., 2002; Nagata et al., 2003), and is of paramount importance to plant evolution (Davies et al., 2012; Schiestl and Johnson, 2013; Sobel and Streisfeld, 2013). Angiosperms exhibit an astonishing polymorphism in flower colors. The phenotypic polymorphism in flower pigmentation is typically manifested in two types: (i) variation in pigment intensity determined by the concentration of pigment, and (ii) variation in floral hue, which is generally determined by the distinction of pigment types or the absence/presence of co-pigments (Wessinger and Rausher, 2012; Sobel and Streisfeld, 2013). In contrast to a wealth of knowledge on the genes related to qualitative variations between different pigment hues and between absence and presence of pigments (Zufall and Rausher, 2003; Hopkins and Rausher, 2011; Wessinger and Rausher, 2012; Sobel and Streisfeld, 2013), very few studies have been performed to characterize the genetic and molecular mechanisms determining the quantitative variation in flower color intensity (Schwinn et al., 2006; Ohno et al., 2013; Wang et al., 2014).

*Paeonia ostii* is a perennial shrub in the genus *Paeonia* and reproduces sexually (Li et al., 2011). It has been reported that *P. ostii* is one of the most important ancestral species of the cultivated tree peony, which is an important ornamental crop in the word and is crowned the “king of flowers” in China (Zhang et al., 2012; Zhou et al., 2014b). Flowers of *P. ostii* exhibit color polymorphism within populations, ranging from nearly white, light pink to deep pink. The pigmentation characteristics of *P. ostii* flowers make them an excellent model for studying the molecular basis of the intensity variation in pigmentation.

In most plant species, flower coloration is primarily caused by flavonoids, particularly anthocyanins (Grotewold, 2006). The anthocyanin biosynthesis pathway is one of the best characterized secondary metabolism pathway in plants, and is highly conserved in structural and regulatory components (Feller et al., 2011; Hichri et al., 2011). Genes encoding enzymes committed to flavonoid biosynthesis, such as chalcone synthase (CHS), chalcone isomerase (CHI), flavanone 3-hydroxylase (F3H), flavonoid 3′-hydroxylase (F3′H), dihydroflavonol reductase (DFR), leucoanthocyanidin dioxygenase (LDOX), and UDP flavonoid glucosyl transferase (UFGT), and genes encoding transporter proteins involved in transportation and storage of floral pigment have been well characterized in many plants (Grotewold, 2006; Chiu et al., 2010; Chen et al., 2011; Zhao et al., 2011; Tanaka and Brugliera, 2013; Li et al., 2014; Zhou et al., 2014a). In addition to the structural components of the pathway, the regulatory mechanisms of the anthocyanin production have also been characterized in several model plants, including petunia (*Petunia hybrida*), snapdragon (*Antirrhinum majus*), *Arabidopsis thaliana* and maize (*Zea mays*) (Cone et al., 1986; Goodrich et al., 1992; Quattrocchio et al., 1998, 1999; Walker et al., 1999; Spelt et al., 2002; Carey et al., 2004; Schwinn et al., 2006; Albert et al., 2011). The anthocyanin biosynthesis genes are mainly activated by an activation complex, consisting of R2R3-MYB, basic helix-loop-helix (bHLH) and WD-repeat (WDR) proteins (MBW complex), at the transcriptional level (Koes et al., 2005; Ramsay and Glover, 2005). Moreover, several repressors that limit the expression of anthocyanin biosynthesis genes have been identified. For instance, *Arabidopsis* R3-MYB protein MYBL2 and *Petunia* R2R3-MYB protein MYB27 can inhibit anthocyanin biosynthesis by forming a MBW inhibitory complex (Kranz et al., 1998; Albert et al., 2014); *Mimulus* R3-MYB factor ROSE INTENSITY1 (ROI1), *Petunia* R3-MYB factor MYBx, *Arabidopsis* R3-MYB factor CAPRICE (CPC) and TRIPTYCHON (TRY), *Arabidopsis* SBP-box protein (SPL9) and JA-ZIM domain proteins can repress the anthocyanin production by inhibiting the formation of MBW activation complex through competing for bHLH or R2R3-MYB partners (Wang et al., 2008; Wester et al., 2009; Zhu et al., 2009; Albert et al., 2011, 2014; Gou et al., 2011; Qi et al., 2011; Yuan et al., 2013); *Arabidopsis* LATERAL ORGAN BOUNDARY DOMAIN (LBD) transcription factors LBD37, LBD38, and LBD39, have also been identified as repressors of anthocyanin biosynthesis (Rubin et al., 2009). However, the information on the regulation of flower pigmentation in tree peonies is lacking, although previous studies have shown that the broad color series in tree peony were primarily determined by the anthocyanin content and types in the petal tissues (Wang et al., 2001; Zhang et al., 2007, 2014; Zhou et al., 2011, 2014a; Zhao et al., 2015). The knowledge from model plants provided useful references for approaching the factors determining flower color intensity in *P. ostii*.

Generally, the variation in anthocyanin concentration is responsible for the flower color intensity (Grotewold, 2006; Tanaka et al., 2008). The quantitative change in anthocyanin intensity is accompanied by the amount alteration of flux through the pathway (Sobel and Streisfeld, 2013). It has been proposed that either increasing the functional activity of pathway enzymes and activation regulators or removing the repressors of anthocyanin production in flowers can result in an increase in anthocyanin intensity (Sobel and Streisfeld, 2013). It is unclear, however, whether intensity variation in flower pigmentation in *P. ostii* is generated by alterations of expression of anthocyanin biosynthesis genes or anthocyanin repressor genes. In this study, we compared the pigment composition and transcriptomes of *P. ostii* flowers with different intensity of coloration. We aimed to explore the correlations between color intensity and anthocyanin concentration, and to identify transcriptional changes and candidate genes potentially responsible for the control of pigmentation intensity in *P. ostii*. The results would provide insights into the molecular basis underlying the intensity variation in flower pigmentation in *P. ostii*.

## Materials and methods

### Plant materials

**Figure 1:**
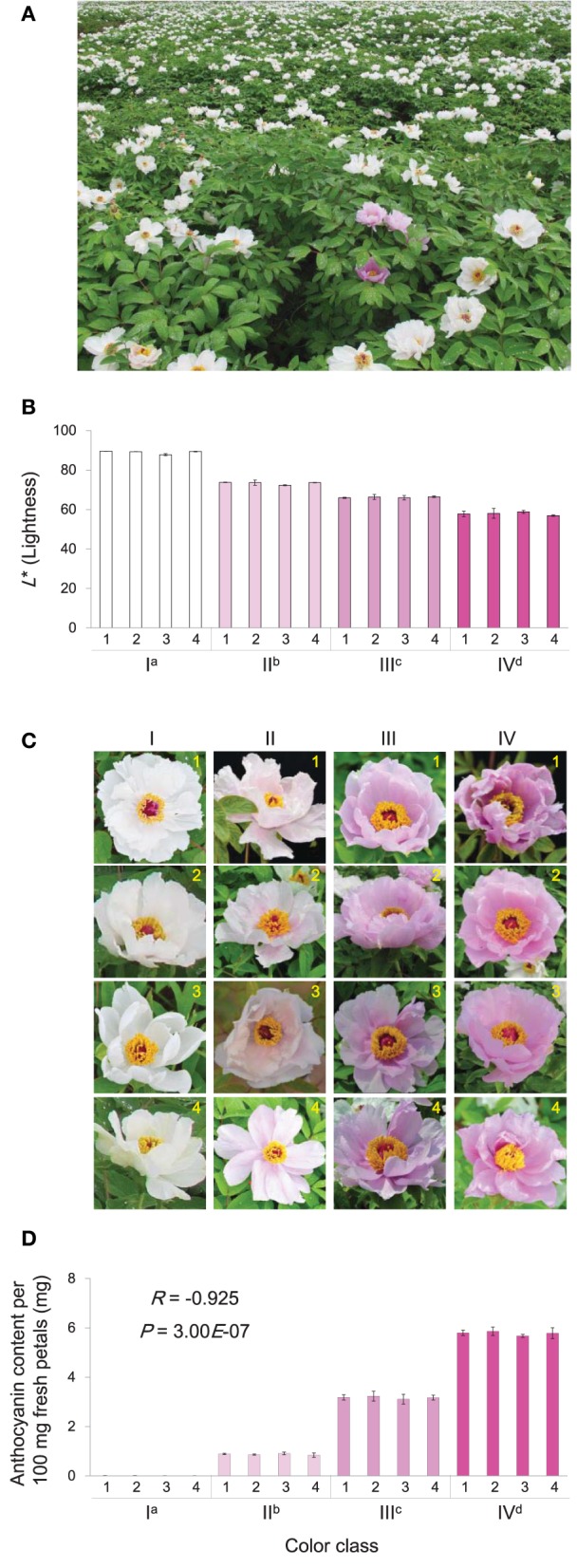
**Characters of ***P. ostii*** flowers used in this study**. **(A)** Typical environment of sampling location. **(B)** The petal L^*^ (Lightness) of flowers in 16 plants belonged to the four color classes (I, II, III, and IV). **(C)** The floral phenotypes of these plants. **(D)** The corresponding anthocyanin concentrations of petals from these plants. In plot **(B,D)**, each value is shown as average ± standard deviation; different superscript letters on the horizontal axis labels indicate statistically significant differences between means of different color classes, as judged by *t*-test (*P* < 0.05). In plot **(D)**, the *R*- and *P*-values indicate the correlation between L^*^ and anthocyanin concentration.

*P. ostii* was grown in the peony planting base of Fenghuangshan, Tongling, Anhui, China (Figure 1A). At full-bloom stage, flower color was analyzed following the International Commission on Illumination (CIE) system. The L^*^ (lightness), a^*^ (redness and greenness), and b^*^ (yellowness and blueness) were measured using a hand-held spectrophotometer (NF333, Nippon Denshoku Industries Co., Ltd., Tokyo, Japan). For each flower, three areas of the medial surface were measured. For each individual plant, the measurement was performed with three petals from three independent flowers. The L^*^ is an indicator of flower color intensity, as lower lightness generally means deeper color. Four classes of color intensity were chosen for this study: L^*^ ranges of >85, 72–75, 65–68, and 57–60 corresponded to color class I, II, III, and IV, respectively. For each color intensity class, four petal samples were collected from different plants and immediately frozen in liquid nitrogen and stored at −80°C until required for anthocyanin analysis and RNA extractions. Each sample contained three to four full-bloom flowers.

### Pigments extraction and high-performance liquid chromatography (HPLC) analyses

Petal samples from 16 plants belonged to four color intensity classes (four plants each) were extracted separately in acidic methanol solution (acetic acid: methanol: water = 1: 4: 5 v/v; 5 ml/100 mg tissue) for 24 h at 4°C and passed through a syringe filter (0.22 μm; Pall). Extracts were analyzed on a Nova Pak C18 column (Waters) and detected using a photodiode array detector (Waters) in the range 190–600 nm. Peonidin-3,5-di-*O*-glucoside (Pn3G5G) and cyanidin-3,5-di-*O*-glucoside (Cy3G5G) were identified using the retention time by comparing with the HPLC data obtained from the commercially available standards (Polyphenols Laboratory, Sandnes, Norway). All determinations were performed with three replicates. Peak area was recorded.

Total anthocyanins and anthoxanthins (flavone and flavonol) were determined semi-quantitatively using the peak area normalization method by employing simple linear regressions using standards Pn3G5G and Cy3G5G for anthocyanins and Rutin for anthoxanthins at 525 and 350 nm, respectively. The content of anthocyanin and anthoxanthin was calculated in mg per 100 mg of fresh petals (as a sum of quantity of Pn3G5G and Cy3G5G mg/100 mg, and as a quantity of Rutin mg/100 mg, respectively).

### RNA extraction and transcriptome sequencing

Total RNA was extracted from the petals using TRIzol Reagent (Life Technologies Corp., Carlsbad, CA, USA) following the manufacturer's standard protocol, then treated with RNase-free DNaseI (Ambion, USA). RNA purity was assessed using a Nanodrop 2000C spectrophotometry (Thermo scientific, USA). Another precipitation step with 0.1 volume of 3 M sodium acetate and 2.5 volumes 100% (vol/vol) ethanol, was conducted if the 260/230 absorbance ratio of the total RNA was less than 1.5. RNA quality was assessed on a 2100 Bioanalyzer (Agilent Technologies, Santa Clara, CA, USA); RNA integrity number (RIN) value was greater than 7.5.

We performed transcriptome sequencing on the bulked RNA of four plants for each flower color intensity class. cDNA library construction and Illumina sequencing were carried out at Beijing Genomics Institute (BGI)-Shenzhen, Shenzhen, China (http://www.genomics.cn/index.php) following the Illimina manufacturer's instructions (Illumina, San Diego, CA, USA). Briefly, poly-A RNA was enriched from 10 μg of total RNA using Magnetic beads with oligo (dT) and broken into short fragments with fragmentation buffer. Using these short fragments as templates, first-strand cDNA was synthesized using random hexamer primer. Then, second-strand cDNA was synthesized using buffer, dNTPs, RNase H (Invitrogen) and DNA polymerase I (Invitrogen). Following size selection and PCR amplification, the cDNA library was sequenced in a HiSeq 2000 to generate paired-end reads. After removing Illumina adapters and reads with unknown nucleotides larger than 5%, and trimming low-quality bases, the remaining high quality reads (clean reads) with an average length of 90 bp were used in this study. The raw sequence data sets were deposited in the US National Center for Biotechnology Information (NCBI) Sequence Read Archive (SRA, http://www.ncbi.nlm.nih.gov/Traces/sra) (Wheeler et al., 2008) under accession number SRP058369, including SRR2027814, SRR2027815, SRR2027817, SRR2027818, SRR2027819, SRR2027820, SRR2027821, and SRR2027822.

### *De novo* assembly and functional annotation

Transcriptome *de novo* assembly was performed with a short reads assembling program—Trinity (Grabherr et al., 2011). We further used a rapid clustering tool—TGICL (Pertea et al., 2003) to assemble unigenes from all four libraries to obtain a single set of non-redundant unigenes. Unigenes were annotated by BLASTX searches against the NCBI non-redundant protein (Nr) database (http://www.ncbi.nlm.nih.gov), Swiss-Prot protein database (http://www.expasy.ch/sprot) and *Arabidopsis* protein database at the Arabidopsis Information Resource (TAIR, http://www.arabidopsis.org) with an *E*-value threshold of 10^−5^. Only the top hit for each sequence was extracted. According to the homology annotation against *Arabidopsis* protein database and NCBI Nr database, gene ontology (GO, http://www.geneontology.org) annotation of unigenes was obtained using Blast2GO program (Conesa et al., 2005). Unigene sequences were also aligned to the Kyoto Encyclopedia of Genes and Genomes Pathway (KEGG; http://www.genome.jp/kegg) database (Ogata et al., 1999) by BLASTx program using an *E*-value cutoff of 10^−5^ to predict the metabolic pathway annotation.

### Digital gene expression profiling and screening of differentially expressed genes

To obtain digital gene expression profiles for different flower color intensity, all of the clean reads from four color classes were separately mapped to the non-redundant reference transcriptome sequences (all-unigenes). SOAPaligner/soap2 (Li et al., 2009) was used for mapping of the reads because of its high alignment accuracy and rapid alignment speed (Hatem et al., 2013; Shang et al., 2014). The number of unambiguously mapped clean reads for each gene in each sample was separately counted. The gene expression level was calculated using the Fragments Per Kilobase of exon per Million fragments mapped (FPKM) method (Mortazavi et al., 2008). Differentially expressed genes were identified based on the method described by Audic and Claverie (1997). The false discovery rate (FDR) was adopted to correct *P*-values in multiple hypothesis tests. A genes was judged to be differentially expressed if it had an FDR = 0.001 and the absolute value of log_2_ Ratio ≥ 2. Unigenes with fewer than 50 reads in all samples were excluded from analysis.

### Go enrichment analysis

GO categories enrichment analysis was implemented using the Bioconductor topGO package (Gentleman et al., 2004; Alexa et al., 2006), with the default arguments and Fisher's exact test to evaluate statistical significance.

### Real-time quantitative reverse transcription polymerase chain reaction (qRT-PCR) validation and expression analysis

qRT-PCR analysis was performed to validate the expression pattern of selected genes identified by the digital expression analysis. The RNA separately extracted from all of the 16 petal samples was used. First strand cDNA was synthesized from 1 μg of total RNA using PrimeScript RT (Perfect Real Time) kit (TAKARA, Japan). The correctness of the gene sequences in the reference transcriptome was validated by reverse transcription PCR and TA cloning using PMD19-T vector kit (TAKARA), followed by sequencing. The qRT-PCR reactions with gene-specific primers (Supplementary Table 1) were performed using a Real-time PCR Detection Systems (RocheCycler 480, Roche, Germany) and SYBRGreen PCR Master Mix (Roche, Germany) according to the manufacturer's instructions. Three independent biological replicates were performed for each reaction. Expression levels of the selected unigenes were normalized to that of an internal reference gene, *glyceraldehyde 3-phosphate dehydrogenase* (*GAPDH*), which was revealed as a stably expressed gene by our Illumina sequencing data and previous studies (Wang et al., 2012). As the PCR efficiency for all the gene-specific primers ranged between 93 and 107% over 1000-fold of cDNA dilution (Supplementary Figure 1 and Supplementary Table 1), relative expression levels were calculated using the 2^−ΔΔCt^ method (Livak and Schmittgen, 2001).

### Phylogenetic analysis

Multiple sequence alignments were produced by ClustalW using default settings, and the phylogenetic trees were created using the neighbor-joining method and bootstrap analysis (1000 replicates) in MEGA5 software (Tamura et al., 2011). The tree in Supplementary Figure 5 was constructed based on an alignment of the R2 and R3 MYB DNA-binding domains of the translated coding sequence of *PoMYB2* (Unigene2047_All) and other MYB sequences. The tree in Supplementary Figure 6 was constructed based on an alignment of the SBP-domains of the translated coding sequence of *PoSPL1* (Unigene24255_All) and other SPL sequences.

## Results

### Flower pigmentation

The typical view of the growth location of *P. ostii* is shown in Figure 1A. Based on the lightness (L^*^) of petals, the flowers of *P. ostii* divided into four classes were chosen for this study. The petals of class I flowers were nearly white, with L^*^ values greater than 85; The class II flowers have light pink petals, with L^*^ values ranging from 72 to 75; The class III flowers have medium pink petals, with L^*^ values ranging from 65 to 68; Deep pink petals were found in the class IV flowers, with L^*^ values ranging from 57 to 60 (Figures 1B,C). Detection of anthocynins present in various flowers showed that the anthocyanin concentration was inversely correlated with L^*^ (Figures 1B,D). A relatively high level of Peonidin-3,5-di-*O*-glucoside (Pn3G5G) and a trace amount of cyanidin-3,5-di-*O*-glucoside (Cy3G5G) were detected in medium and deep pink flowers. A low level of Pn3G5G was detected in the light pink flower, while Cy3G5G was almost undetectable. Both Pn3G5G and Cy3G5G were undetectable in the white flower (Supplementary Figure 2). Pn3G5G and Cy3G5G are synthesized based on peonidin and cyanidin, respectively. Cyanidin and peonidin are two similar anthocyanidins derived from the same branch of the anthocyanin biosynthesis pathway, differing in that peonidin has a methyl substitution on the B-ring.

### Transcriptome *de novo* assembly and functional annotation

In order to identify genes associated with flower color variation in *P. ostii*, we separately sequenced the petal transcriptomes of flowers of different color classes. *De novo* assembly using approximately 129, 138, 130, and 139 million paired-end clean reads generated 59,532, 58,169, 60,929 and 60,896 unigenes in class I, II, III, and IV, respectively (Table 1). A total of 66,501 non-redundant all-unigenes were obtained after clustering, with average length and N50 (50% of the assembled bases are incorporated into contigs of length N or larger) of 973 and 1475 bp respectively (Table 1). Among 66,501 all-unigenes, 41,409 (62.27%) were larger than 500 bp in length (Supplementary Table 2). The annotation results of all-unigenes were shown in Supplementary Table 3.

**Table 1:** **Summary of transcriptome sequencing and assembly results in ***P. ostii*****.

	**Color class**				**All-unigene**
	**I**	**II**	**III**	**IV**	
Total raw reads	135,116,924	145,233,346	136,521,718	145,463,452	
Total clean reads	128,894,136	138,149,888	130,132,618	138,888,284	
Total clean nucleotides (nt)	11,600,472,240	12,433,489,920	11,711,935,620	12,499,945,560	
Q20 percentage	98.09%	98.02%	97.98%	98.12%	
N percentage	0.00%	0.00%	0.00%	0.00%	
GC percentage	44.13%	44.62%	44.01%	44.10%	
**CONTIG**					
Total number	86,541	84,919	87,972	86,754	
Total length(nt)	39,098,528	38,556,647	40,750,287	40,298,025	
Mean length(nt)	452	454	463	465	
N50	1011	1022	1054	1048	
**UNIGENE**					
Total number	59,532	58,169	60,929	60,896	66,501
Total length(nt)	44,759,351	43,506,064	47,184,533	47,622,382	64,718,581
Mean length(nt)	752	748	774	782	973
N50	1323	1312	1360	1359	1475
Total consensus sequences	59,532	58,169	60,929	60,896	66,501
Distinct clusters	17,510	16,784	18,287	18,359	27,934
Distinct singletons	42,022	41,385	42,642	42,537	38,567

**Figure 2:**
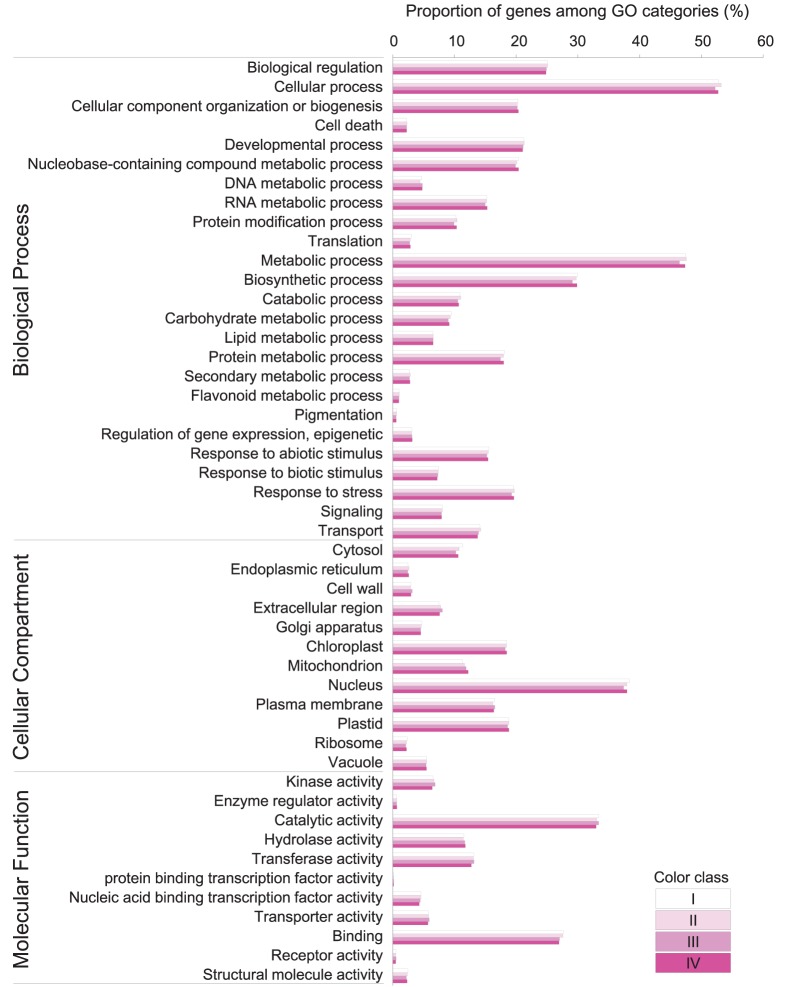
**Gene classification based on GO for unigenes in ***P. ostii*** of different flower color classes**. The results are summarized in three main categories: biological process, cellular compartment, and molecular function. The frequency of GO terms was analyzed using GO Slim Assignment. Bars indicate the ratio of each cluster.

Based on BLAST, 33,289 (55.92%), 33,130 (56.95%), 34,067 (55.91%), and 34,188 (56.14%) unigenes in class I, II, III, and IV respectively, showed significant similarity to known proteins (Table 2). 29,621, 29,433, 30,213, and 30,293 unigenes could be assigned to at least one Gene Ontology (GO) term in class I, II, III, and IV, respectively. Base on the GO term annotation, the unigenes were categorized into 48 functional groups (Figure 2). The distribution patterns of unigenes from the four classes were similar under different GO categories (Figure 2). Unigenes assigned to categories of “cellular process,” “catalytic activity,” and “metabolic process” were dominant. More than 470 unigenes were assigned to “flavonoid metabolic process” and “pigmentation” categories in each class of flowers. KEGG pathway analyses showed that 18,130, 18,083, 18,637, and 18,770 unigenes in class I, II, III, and IV respectively, could be assigned to 128 KEGG pathways. The most represented pathways were “metabolism” (containing around 21% unigenes in each class), “biosynthesis of secondary metabolites” (around 11% unigenes in each class) and “plant hormone signal transduction” (around 5% unigenes in each class) (Supplementary Table 4).

**Table 2:** **Number of unigenes of ***P. ostii*** annotated with various databases**.

	**Color class**				**All-unigene**
**Database**	**I**	**II**	**III**	**IV**	
Nr	31,668	31,541	32,346	32,454	36,579
Swiss-Prot	20,091	19,966	20,763	20,735	23,876
KEGG	18,130	18,083	18,637	18,770	21,929
TAIR	28,985	28,716	29,527	29,597	33,772
GO	29,621	29,433	30,213	30,293	34,141
Total	33,289	33,130	34,067	34,188	38,658

### Differentially expressed unigenes between flowers with varied color intensities

**Figure 3:**
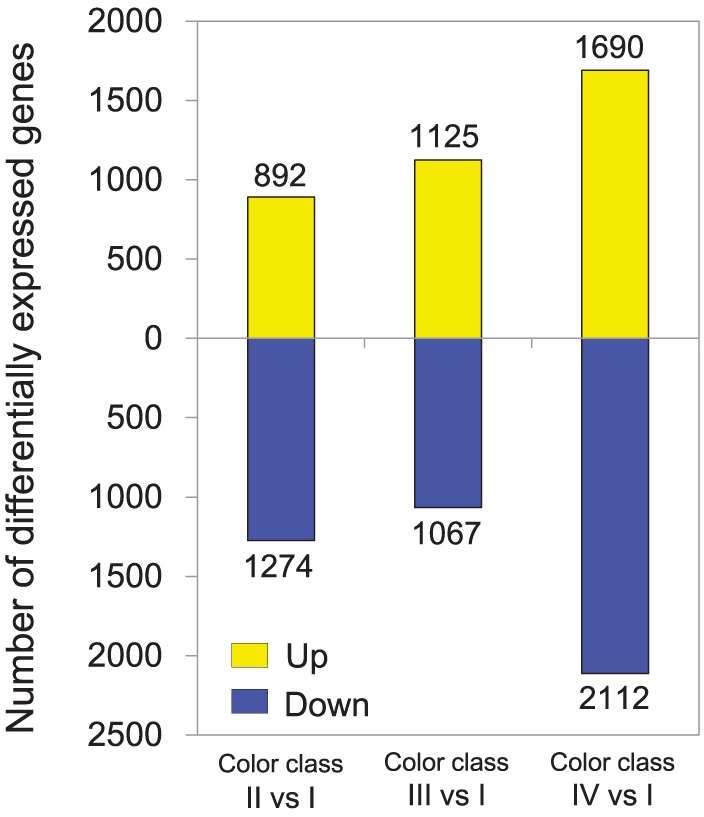
**Changes in gene expression profile between ***P. ostii*** petals of different color classes**. The number of up- and down-regulated genes between class II and class I, class III and class I, and class IV and class I are summarized. For example, “892” means the expression of 892 genes was significantly higher in color class II than in color class I; and “1274” means the expression of 1274 genes was significantly lower in color class II than in color class I.

Gene expression profiles of petals were compared between flowers with different color intensities. 55.50, 57.28, 55.27, and 55.47% reads of class I, II, III and IV libraries were aligned uniquely to the reference transcriptome obtained via combined assembly. A total of 7187 unigenes were differentially expressed between flowers with different color intensities (Supplementary Table 5). Most of the differentially expressed unigenes were found between pink and white flowers. The highest number of differentially expressed unigene was observed between flowers of class I and IV (Figure 3). One hundred and twenty-eight unigenes were found to be commonly up-regulated in various pink flowers, while 424 unigenes being commonly down-regulated. To validate our expression data obtained by RNA sequencing, the differential expression patterns of 19 unigenes were verified by qRT-PCR (Supplementary Figure 3). A high correlation was observed between expression levels obtained by RNA-Seq and qRT-PCR (*R* = 0.787, *P* = 1.90*E*-13) (Supplementary Figure 3).

**Figure 4:**
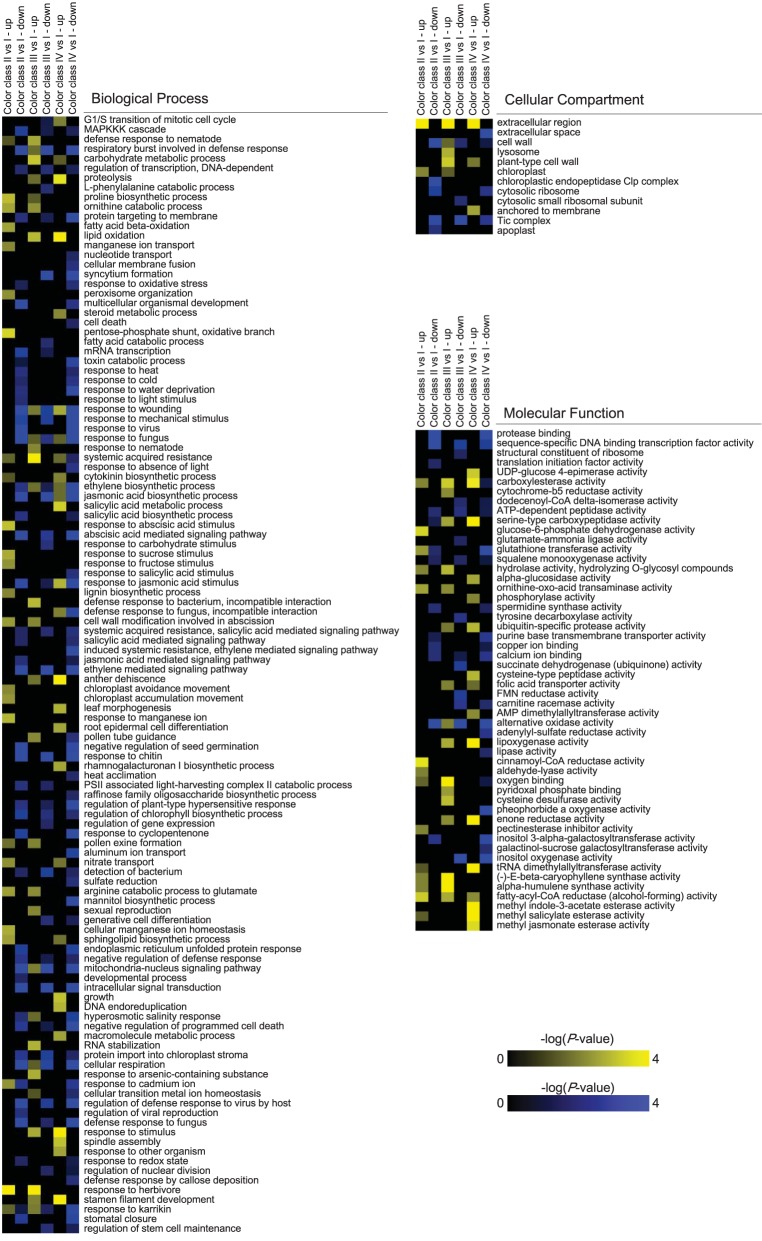
**Overrepresented GO terms amongst differentially expressed genes between ***P. ostii*** petals of different color classes**. Only GO terms are shown where *P* < 0.01 in at least one of the six gene sets (up- or down-regulated in any of these three comparisons: class II vs. class I, class III vs. class I, or class IV vs. class I).

GO enrichment was carried out on differential expressed genes (Figure 4). The GO categories enriched in the up-regulated gene set include “Fatty acid beta-oxidation,” “extracellular region,” “carboxylesterase activity,” and “fatty-acyl-CoA reductase activity.” Several categories, such as “MAPKKK cascade,” “intracellular signal transduction,” “ethylene mediated signaling pathway,” “ATP-dependent peptidase activity,” “squalene monooxygenase activity,” and “calcium ion binding,” were significantly enriched in the down-regulated gene set.

**Figure 5:**
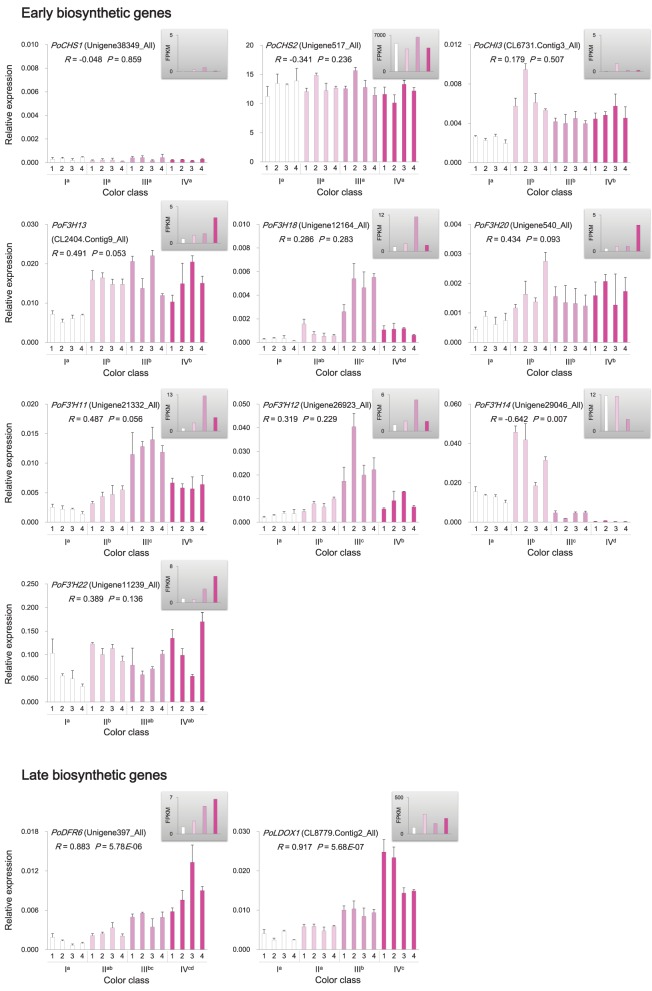
**Expression of anthocyanin structural genes in ***P. ostii*** of different flower color classes**. The expression was investigated by qRT-PCR (shown in the bigger histograms) and RNA-seq (shown in the smaller histograms in the gray shadow areas) of petal samples from the four color classes (I, II, III, and IV). The analyzed plants were shown in Figure 1. For qRT-PCR, each value was normalized relative to *PoGAPDH* expression and is shown as average ± standard deviation from three biological replicate sampling. Different superscript letters on the horizontal axis labels indicate statistically significant differences between means of different color classes, as judged by *t*-test (*P* < 0.05). The *R*- and *P*-values given in the plot indicate the correlation between anthocyanin concentration and gene expression. CHS, chalcone synthase; CHI, chalcone isomerase; F3H, flavanone 3-hydroxylase; F3′H, flavonoid 3′-hydroxylase; DFR, dihydroflavonol 4-reductase; LDOX, leucoanthocyanidin dioxygenase.

**Figure 6:**
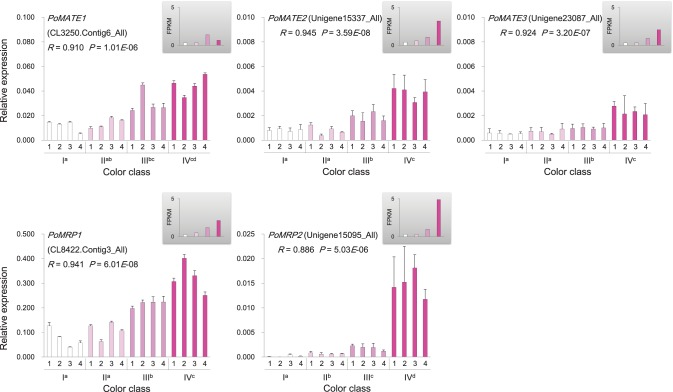
**Expression of anthocyanin transporter genes in ***P. ostii*** of different flower color classes**. The expression was investigated by qRT-PCR (shown in the bigger histograms) and RNA-seq (shown in the smaller histograms in the gray shadow areas) of petal samples from the four color classes (I, II, III, and IV). The analyzed plants were shown in Figure 1. For qRT-PCR, each value was normalized relative to *PoGAPDH* expression and is shown as average ± standard deviation from three biological replicate sampling. Different superscript letters on the horizontal axis labels indicate statistically significant differences between means of different color classes, as judged by *t*-test (*P* < 0.05). The *R*- and *P*-values given in the plot indicate the correlation between anthocyanin concentration and gene expression. MATE, multidrug and toxin extrusion transporter; MRP, ATP binding cassette transporter.

Genes involved in anthocyanin biosynthesis and transport were identified from the *P. ostii* transcriptome (Supplementary Figure 4 and Supplementary Table 6), and their expression levels in different classes of flowers were measured by RNA-Seq and qRT-PCR. The results showed that genes encoding chalcone isomerase (CHI; *PoCHI3*), flavanone 3-hydroxylase (F3H; *PoF3H13, PoF3H18*, and *PoF3H20*), flavonoid 3′-hydroxylase (F3′H; *PoF3*′*H11*, and *PoF3*′*H12*), dihydroflavonol reductase (DFR; *PoDFR6*), and leucoanthocyanidin dioxygenase (LDOX; *PoLDOX1*) were expressed at a higher level in various pink flowers than in white ones (Figure 5). Notably, the expression levels of *PoDFR6* and *PoLDOX1* were well correlated with flower color intensity and anthocyanin concentration (Figure 5). These two genes were involved in the later steps of anthocyanin biosynthesis. The expression of genes encoding chalcone synthase (CHS; *PoCHS1*, and *PoCHS2*) and UDP flavonoid glucosyl transferase (UFGT; *PoUFGT1, PoUFGT2, PoUFGT3, PoUFGT4*, and *PoUFGT5*) showed no significant difference between pink and white flowers (Figure 5, Supplementary Table 6). The anthocyanins are stored in the central vacuole in plants (Kitamura, 2006). The activity of vacuolar flavonoid transporters, such as multidrug and toxin extrusion (MATE) and ATP-binding cassette (ABC) transporters, can affect anthocyanins accumulation in vacuole, which in turn affects the intensity of pigmentation in plant tissues (Goodman et al., 2004; Gomez et al., 2009; Zhao et al., 2011). The increasing tendency for anthocyanin accumulation in pink flowers of *P. ostii* was also accompanied by increased expression of genes encoding MATE transporters (*PoMATE1, PoMATE2*, and *PoMATE3*) and ABC transporters (*PoMRP1* and *PoMRP2*) (Figure 6), suggesting their potential roles in promoting anthocyanins accumulation in *P. ostii*.

**Figure 7:**
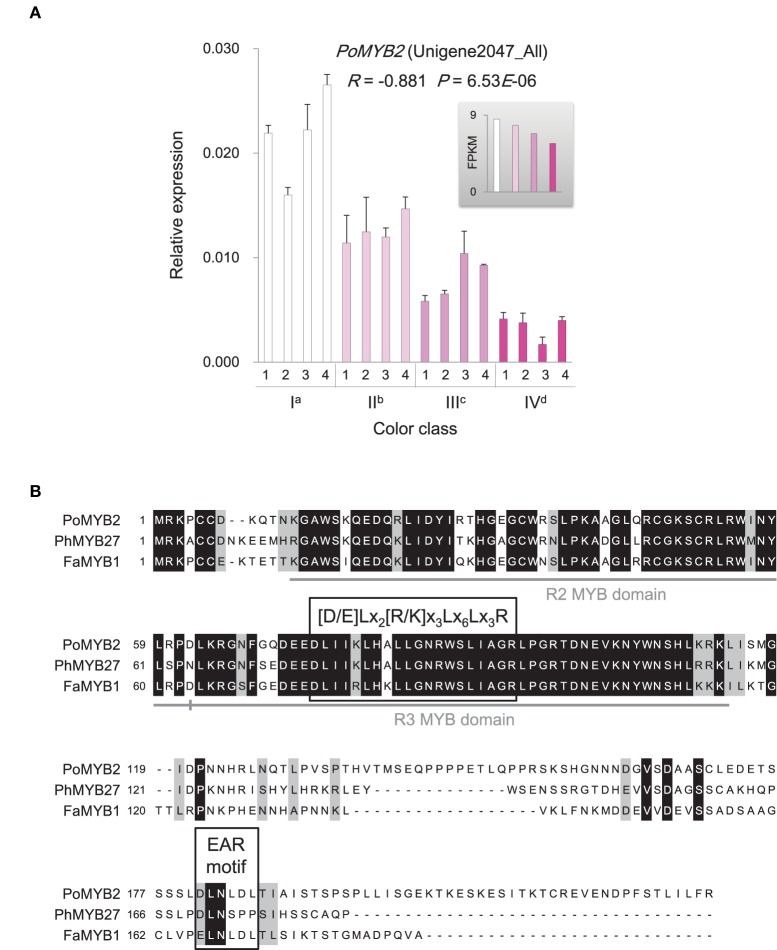
**Expression and sequence similarity analysis of ***PoMYB2*****. **(A)** Expression of *PoMYB2* gene in *P. ostii* of different flower color classes. The expression was investigated by qRT-PCR (shown in the bigger histogram) and RNA-seq (shown in the smaller histogram in the gray shadow area) of petal samples from the four color classes (I, II, III, and IV). The analyzed plants were shown in Figure 1. For qRT-PCR, each value was normalized relative to *PoGAPDH* expression and is shown as average ± standard deviation from three biological replicate sampling. Different superscript letters on the horizontal axis labels indicate statistically significant differences between means of different color classes, as judged by *t*-test (*P* < 0.05). The *R*- and *P*-values given in the plot indicate the correlation between anthocyanin concentration and gene expression. **(B)** Amino acid sequence alignments of PoMYB2, PhMYB27, and FaMYB1. The bHLH interaction motif ([D/E]Lx_2_[R/K]x_3_Lx_6_Lx_3_R) and EAR repressor motif are boxed. Identical amino acids are indicated in black, similar amino acids in gray, gaps introduced in the alignment are indicated with dashes. The full-length coding sequence of *PoMYB2* is displayed in Supplementary Data Sheet 1.

**Figure 8:**
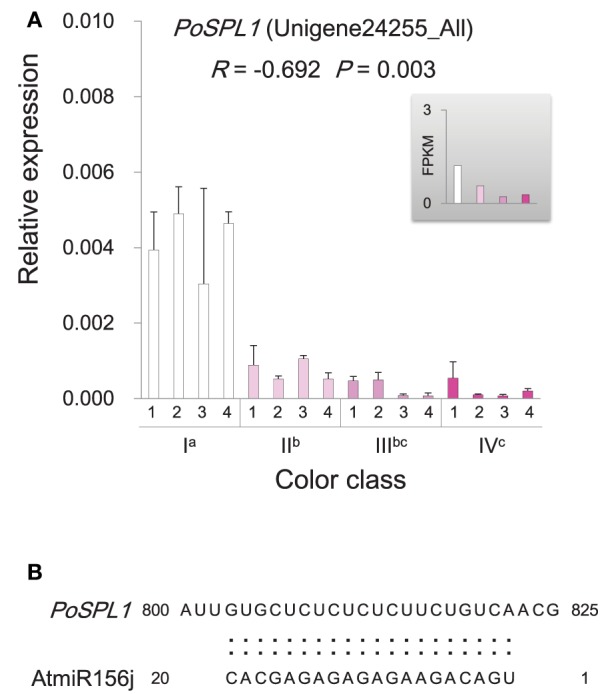
**Expression and sequence characteristics of ***PoSPL1*****. **(A)** Expression of *PoSPL1* gene in *P. ostii* of different flower color classes. The expression was investigated by qRT-PCR (shown in the bigger histogram) and RNA-seq (shown in the smaller histogram in the gray shadow area) of petal samples from the four color classes (I, II, III, and IV). The analyzed plants were shown in Figure 1. For qRT-PCR, each value was normalized relative to *PoGAPDH* expression and is shown as average ± standard deviation from three biological replicate sampling. Different superscript letters on the horizontal axis labels indicate statistically significant differences between means of different color classes, as judged by *t*-test (*P* < 0.05). The *R*- and *P*-values given in the plot indicate the correlation between anthocyanin concentration and gene expression. **(B)** The conserved miR156 target site in the coding sequence of *PoSPL1*. Alignment of the AtmiR156 sequence with *PoSPL1*. The full-length coding sequence of *PoSPL1* is displayed in Supplementary Data Sheet 1.

The MBW ternary protein complex composed of R2R3-MYB and basic helix-loop-helix (bHLH) transcription factors as well as WD-repeat (WDR) proteins has been documented as a primary regulator in anthocyanin biosynthesis (Heim et al., 2003; Baudry et al., 2004; Zimmermann et al., 2004; Koes et al., 2005; Ramsay and Glover, 2005; Lepiniec et al., 2006; Petroni and Tonelli, 2011). However, genes encoding the active MBW complex components were not significantly differentially expressed among flowers with different color intensities. Instead, a gene, *PoMYB2*, was identified to exhibit an inverse relationship between its expression and anthocyanin accumulation (Figure 7A). Phylogenetic analysis showed that *PoMYB2* was homologous to *Petunia PhMYB27* and strawberry *FaMYB1* (Supplementary Figure 5), which have been suggested to act as transcriptional repressors of anthocyanin accumulation (Aharoni et al., 2001; Albert et al., 2011). A well-conserved bHLH interaction motif [D/E]Lx_2_[R/K]x_3_Lx_6_Lx_3_R (Zimmermann et al., 2004) was found in the R3 repeat domain in the predicted protein sequence of *PoMYB2* (Figure 7B). A putative repression motif LNLDL conforming to the LxLxL type of ERF-associated amphiphilic repression (EAR) motif was present in the C terminal region of the predicted PoMYB2 protein sequence (Kagale and Rozwadowski, 2011) (Figure 7B). In addition to *PoMYB2, PoSPL1*, a gene homologous to the miR156-targeted *Arabidopsis* SPL protein AtSPL13 (Guo et al., 2008; Wu et al., 2009), also exhibited a negative relationship between its expression and anthocyanin concentration (Figure 8A, Supplementary Figure 6). A conserved miR156 target site in the coding sequence of *PoSPL1* was also identified (Figure 8B, Supplementary Data Sheet 1).

## Discussion

Anthocyanin concentration was closely correlated with the intensity of petal color in *P. ostii*. Anthocyanins were synthesized via the flavonoid pathway. The corresponding genes have been well characterized and been divided into early biosynthetic genes and late biosynthetic genes in dicotyledonous plants (Martin and Gerats, 1993; Mol et al., 1998; Petroni and Tonelli, 2011). The early biosynthetic genes, including *CHS, CHI, F3H*, and *F3*′*H*, located upstream of the anthocyanin biosynthetic pathway and led to the production of flavonols and other flavonoid compounds, while the late biosynthetic genes, including *DFR* and *LDOX*, were downstream genes in the anthocyanin biosynthetic pathway and were specifically for anthocyanin biosynthesis (Lepiniec et al., 2006; Petroni and Tonelli, 2011). In *P. ostii*, the late anthocyanin biosynthetic genes were differentially expressed among flowers with different color intensities, displaying a strong correlation between their expression levels and the concentration of anthocyanin in petals, whereas none of the early biosynthetic genes exhibited expression patterns significantly positively correlated with the concentration of anthocyanin (Figure 5). The increased expression of *DFR* (*PoDFR6*) in deeper color flowers probably led more dihydroflavonols into the direction of anthocyanin branch (Figure 5, Supplementary Figure 4). In contrast, the higher expression of genes encoding flavonol synthase (*PoFLS1*: CL8054.Contig1_All and *PoFLS4*: Unigene22443_All) and flavonol 3-O-glucosyltransferase (CL4664.Contig3_All) in the nearly white flowers probably promoted the conversion of dihydroflavonols to flavonols (Supplementary Table 6 and Supplementary Figure 4), resulting in the relatively higher accumulation of anthoxanthins in the nearly white flower than in deeper color flowers (Supplementary Figure 2). Previous studies have shown that inactivation of FLS promoted the accumulation of anthocyanin, while inactivation of DFR promoted the accumulation of flavonols (Pelletier et al., 1999; Owens et al., 2008; Stracke et al., 2009). Substrate competition seems to exist between FLS and DFR, controlling the metabolic flux through the branches of the flavonoid biosynthetic pathway underlying flower color intensity variation in *P. ostii*. A significant positive correlation was also observed between the expression levels of anthocyanin transporter genes and the anthocyanin levels in petals of different flowers (Figure 6). On the contrary, the putative anthocyanin–biosynthesis repressor genes were expressed at a high level in the nearly white petals and gradually reduced in deep color flowers, exhibiting an expression pattern inverse to anthocyanin biosynthetic and transporter genes (Figures 7, 8). The coordinated expression of genes involved in anthocyanin biosynthesis, anthocyanin transport and suppression of anthocyanin synthesis suggested a sophisticated regulatory system underlying anthocyanin pigmentation in *P. ostii*.

It has been revealed that the combinatorial action of R2R3-MYB and bHLH transcription factors, along with WDR proteins, played an important role in regulating the transcription of anthocyanin genes (Heim et al., 2003; Baudry et al., 2004; Zimmermann et al., 2004; Koes et al., 2005; Ramsay and Glover, 2005; Lepiniec et al., 2006; Petroni and Tonelli, 2011; Thévenin et al., 2012; Xu et al., 2014). The action of the MBW complex is primarily specified by the activity of R2R3-MYB factors in the complex. Diverged members of the R2R3-MYB gene family had distinct roles in determining the action of the complex either to promote or inhibit the transcription of anthocyanin biosynthesis genes (Matsui et al., 2008). In *P. ostii*, genes homologous to the subunits of the active MBW complex were not differently expressed in flowers with different color intensity. Instead, a new R2R3-MYB gene encoding PoMYB2 highly similar to PhMYB27 (Figure 7B, Supplementary Figure 5) which can reduce anthocyanin production either by repressing the expression of key factors required for MBW complex thus preventing the MBW complex formation or by incorporating to MBW activation complexes to convert them into repressive complexes (Albert et al., 2014), was highly expressed in the nearly white flower and gradually reduced expression in flowers with increasing color intensity (Figure 7A). In line with the inverse expression pattern of *PoMYB2* with that of the late anthocyanin biosynthetic genes, the PoMYB2 protein contains an amino acid motif [D/E]Lx_2_[R/K]x_3_Lx_6_Lx_3_R, which is required for interaction with bHLH partners in MBW complex (Zimmermann et al., 2004), and a C terminal EAR repressor motif (LNLDL), which mediate transcriptional repression in plants (Kagale and Rozwadowski, 2011) (Figure 7B). A similar pattern of expression was also observed for the gene *PoSPL1*, which was homologous to *AtSPL13* (Figure 8A, Supplementary Figure 6). Although the functional evidence on AtSPL13 is lacking to date, previous studies have shown that *AtSPL13* is one of the miR156-targeted SPL genes, which also include *AtSPL3, AtSPL9*, and *AtSPL10* (Guo et al., 2008; Wu et al., 2009). Previous functional analyses revealed that the miR156-targeted SPLs shared some common regulatory functions (Yu et al., 2010). For example, both AtSPL9 and AtSPL13 are involved in the regulation of trichome production (Yu et al., 2010; Preston and Hileman, 2013). A well-conserved miR156 target site was also present in the coding sequence of *PoSPL1* (Figure 8B). It has been confirmed that *AtSPL9* can repress anthocyanin production through its “squelching activity” causing the depletion of transcription factors essential for activation (Gou et al., 2011). That is, AtSPL9 can serve as a competitive inhibitor that acts by binding R2R3-MYB proteins required for the formation of MBW complex for regulation of anthocyanin production (Gou et al., 2011). The expression pattern of *PoSPL1* exhibited a negative correlation with the anthocyanin concentration (Figure 8A), implying a possible role of PoSPL1 in repressing anthocyanin synthesis in *P. ostii*. All these findings suggested the existence of a complex regulatory network controlling anthocyanin synthesis in *P. ostii*. Negative regulators identified in *P. ostii* seem to be the major factor responsible for the intensity variation of *P. ostii* in anthocyanin pigmentation. It is likely that decreased expression of *PoMYB2* and *PoSPL1* released the repression of anthocyanin structural genes, that in turn increased the flower color intensity in *P. ostii*.

The decreased expression of *PoMYB2* and *PoSPL1* in deeper color flowers was unlikely caused by loss-of-function mutations because we did not find critical differences in the coding sequences of *PoMYB2* and *PoSPL1* from different intensity variants. Additionally, these two genes belong to different gene families and function through distinct mechanisms. The probability is small for *PoMYB2* and *PoSPL1* to mutate simultaneously to result in a coordinated decrease in expression in *P. ostii*. *PoMYB2* and *PoSPL1* might be coordinately regulated by other elements. Gene expression profiling showed that many genes involved in MAPKKK cascade, respiratory burst, phytohormone biosynthesis, and various organic substance metabolic processes were differentially expressed among different intensity variants (Figure 4). The expression levels of two genes (Unigene12751_All, Unigene10302_All) encoding sucrose synthases were positively correlated with the flower color intensity (Supplementary Table 5). Sucrose can promote the anthocyanin production by suppressing the expression of the transcription factor *MYBL2* which negatively regulates anthocyanin biosynthesis, while concurrently inducing the expression of the positive regulators, including *PAP1, TT8*, and *GL3* (Solfanelli et al., 2006; Jeong et al., 2010; Das et al., 2012). Previous studies in *Arabidopsis* showed that the accumulation of anthocyanin in leaves is positively correlated with the increase in endogenous sucrose content (Jeong et al., 2010). Genes involved in ethylene mediated signaling pathway were down-regulated in deeper color flowers (Figure 4), that is of particular interest because it has been revealed that ethylene plays a negative regulatory role in anthocyanin biosynthesis and functions by regulating the activity of positive and negative regulators at the transcriptional level (Jeong et al., 2010; Das et al., 2011, 2012; Qi et al., 2011). Ethylene also has a suppression effect on the sucrose-induced anthocyanin pigmentation (Jeong et al., 2010). *Arabidopsis* mutants defective in ethylene signaling or wild-type plants treated with ethylene biosynthesis and ethylene-binding inhibitors displayed a promotion on anthocyanin accumulation (Jeong et al., 2010). The alteration of the internal environment resulted from changes in sucrose and ethylene synthesis and signaling probably had an effect on transcriptional variation of anthocyanin repressor genes in different color variants. Fatty acid beta-oxidation was also found among the enriched GO categories in the up-regulated gene sets in deeper color classes (Figure 4). The up-regulation patterns of genes encoding acyl-CoA oxidase (*PoACX*, Unigene12172_All) and peroxisomal 3-ketoacyl-CoA thiolase (*PoPKT*, Unigene29334_All, Unigene33907_All) were coordinated with the increase in flower color intensity (Supplementary Table 5). This result probably has implications for the re-allocation of acyl-CoA between fatty acid and anthocyanin biosynthesis. As the final product of fatty acid beta-oxidation, acyl-CoA can be used as a substrate in anthocyanin biosynthesis. The induction of enzymes involved in fatty acid beta-oxidation may result in split-flowing of acyl-CoA and eventually enhance the production of anthocyanin in deeper color petals.

Tree peony is an important ornamental crop in the word, especially in East Asia. Classical breeding techniques have enabled the creation of a wide range of flower colors for tree peony cultivars. It has been look forward, however, to produce more varieties with desirable and novel flower colors by genetic engineering. *P. ostii* is one of the most important ancestral species of the cultivars of tree peony. The results of this study provided deep insights into the molecular basis underlying the variation in flower color intensity in tree peony, providing a foundation for developing novel flower colors through manipulating the anthocyanin biosynthesis pathway.

## Conclusions

Flowers of *P. ostii* exhibited variations in color intensity, which was significantly correlated with the anthocyanin concentration. Genome-wide transcription analysis showed that, although anthocyanin biosynthesis had a direct effect on the pigmentation of *P. ostii* flowers, other metabolic and hormone-mediated signaling pathways were also contributed to the color intensity variation in *P. ostii*. Differential expression of genes encoding anthocyanin repressors, which negatively regulate the expression of anthocyanin biosynthesis genes by affecting the activation capacity of the MYB-bHLH-WDR complex, seems to be the major factor responsible for the intensity variation in anthocyanin pigmentation in *P. ostii*. This study provided insights into the potential key components related to the regulation of flower color intensity in *P. ostii*. As *P. ostii* is one of the most important ancestral species of the cultivars of tree peony, these results might provide a foundation for developing novel flower colors in ornamental cultivars through manipulating the anthocyanin biosynthesis pathway.

## Author contributions

LG designed the research, performed the experiments and the data analysis, and drafted the manuscript. HY contributed analysis tools and participated in the data analysis and manuscript preparation. HL helped in the HPLC experiments and data analysis; JY participated in the design of the study, helped in data analysis and manuscript preparation. YH conceived the idea, and participated in the design of the study and in interpreting results, and manuscript preparation. All authors carefully read and approved the final manuscript.

## Funding

This project was supported by the Chenshan Key Scientific Research Projects (F122431, G142421), and Key Technologies R&D Program of Shanghai (14DZ2260400).

### Conflict of interest statement

The authors declare that the research was conducted in the absence of any commercial or financial relationships that could be construed as a potential conflict of interest.

## Abbreviations

Pn3G5G: peonidin-3,5-di-O-glucoside
Cy3G5G: cyanidin-3,5-di-O-glucoside
PAL: phenylalanine ammonia-lyase
C4H: cinnamate 4-hydroxylase
4CL: 4-coumaroyl:CoA ligase
CHS: chalcone synthase
CHI: chalcone isomerase
F3H: flavanone 3-hydroxylase
F3′H: flavonoid 3′-hydroxylase
FLS: flavonol synthase
DFR: dihydroflavonol 4-reductase
LDOX: leucoanthocyanidin dioxygenase
UFGT: UDP flavonoid glucosyl transferase
MATE transporter: multidrug and toxin extrusion transporter
MRP transporter: ATP binding cassette transporter
bHLH: basic helix-loop-helix
WDR: WD-repeat protein
SPL: SBP-box transcription factor
FDR: False discovery rate
FPKM: Fragments per kilobase of exon per million fragments mapped
GO: Gene Ontology
RIN: RNA integrity number.
