# Supplementary material for: Extensive Transcriptome Changes Underlying the Flower Color Intensity Variation in Paeonia ostii
Source: Front Plant Sci. 2016 Jan 6;6:1205. doi: 10.3389/fpls.2015.01205 (PMC4702479; doi:10.3389/fpls.2015.01205)
Supplement: Supplementary file 7 [file DataSheet1.DOCX]

**Supplementary Data Sheet 1.** Coding sequences of *PoMYB2* and *PoSPL1*.

>PoMYB2

ATGAGAAAACCTTGCTGTGATAAACAAACCAACAAGGGAGCCTGGTCTAAACAAGAAGACCAAAGGCTCATCGATTACATCCGAACTCATGGGGAAGGCTGTTGGCGTTCCCTCCCAAAGGCTGCAGGGTTGCAACGTTGTGGTAAAAGTTGCAGACTGAGATGGATAAACTATCTCAGACCAGACCTCAAACGAGGCAACTTTGGTCAAGATGAAGAGGATCTCATCATCAAGCTCCATGCACTCCTTGGTAACAGGTGGTCATTGATAGCAGGAAGGTTGCCTGGAAGAACAGACAATGAGGTAAAGAACTATTGGAACTCTCATCTTAAGAGAAAACTAATAAGCATGGGTATTGACCCTAATAACCATCGCCTGAATCAAACTCTTCCCGTTTCCCCCACCCATGTTACAATGTCGGAGCAACCTCCGCCTCCAGAGACACTGCAGCCACCACGGTCAAAATCACATGGCAATAACAATGATGGGGTGTCAGACGCTGCTAGTTGCCTTGAAGACGAGACATCCAGCAGCTCGCTTGACTTGAATCTTGATCTTACCATTGCCATTTCTACTTCTCCTTCACCACTTCTAATTTCTGGCGAAAAGACAAAAGAAAGTAAGGAGTCCATAACAAAAACATGCAGGGAAGTCGAAAATGACCCGTTTTCGACTCTTATTCTTTTTAGATAG

>PoSPL1

ATGGACTGGAATTTGATGGCATCTGCCTGGGACTTACCTGAATTGGTCCGGGAAGAGGACACCCCCCTTGCTGCTCTTGTTGGATCAAGTAGCTTGGGGATGCATAAGAATAAAGGAGATTTTTCAGTTGATTTGAAACTGGGGAGATTGGGTGATTTAGGAGACAAATCAGTGAGCAAATTGAAGGACCCAAAGCCGTCAACAGCAGTTTCATCTCCCTCTGGGCCATCAAAGAAGGCTCGTACCTTGACTCTTCATGTGTCTTGTTTGGTTGATGGGTGTACTGCCGATCTTAGCAAGTGCAGGGAATACCATCGGCGCCATAGGGTCTGCGAGCGCCATTCCAAGACCCCGACTGTGACCGTCCGAGGGGAAGAGCAACGCTTCTGCCAGCAATGCAGCAGATTTCATTCTCTCGGGGAATTCGATGACGTAAAGAGAAGCTGCCGGAAACGTCTTGATGGGCACAATCGCCGCCGAAGAAAGCCTCAACAACCACCCCTTCGAATGAATTCAGGCAACGTTCTTTCCAACTACCAAGGATTCGAGTACAATCGACACCAACAGTTGCAAGATATAAAACCAGCATGCCTTCCAGGTCCATCCTTCTCTTACGTCTACAACGGAGGAAACGAAAGGTTCATGCAGGCCAATGACCTTAAGATGGGCAAGCGTACAACTCCTCACGAAGCTCCTTCAGTCTGCCAGCCACTGCTCCACAACATAGCATCTCCAGAAAGTTTCAGGGGGAACCATAAAATGCTATCAAGTAATGGGTTATATCAATCTGTTGATTCAGATT**GTGCTCTCTCTCTTCTGTCA**ACGCATCCAAAGCAATCTTCGGGAGGGTTTGGTTTGAACAACATGTTGCAGTCCAATGTGTCTCTTCCTCCCGCACGGCCAATCCTAGATTCTGGGCTGCATTTTAATAACATGGCTCAGTACTCATGCTCACTTGGCATGGCGGGAGAGCCGGTGGACTTAATCCCTGGTGGCAGCAACAACACCAACATGCAACGGGGCGGGATGTTTCATGGGTGGCCTAATTGCATTCCAAAAAATTTCCATTTTCTTGGAAGTAGCTAA
