# Supplementary figures and images for: Extensive Transcriptome Changes Underlying the Flower Color Intensity Variation in Paeonia ostii
Source: Front Plant Sci. 2016 Jan 6;6:1205. doi: 10.3389/fpls.2015.01205 (PMC4702479; doi:10.3389/fpls.2015.01205)

**Supplementary Figure 1.** Amplification efficiencies of the primers used for qRT-PCR.

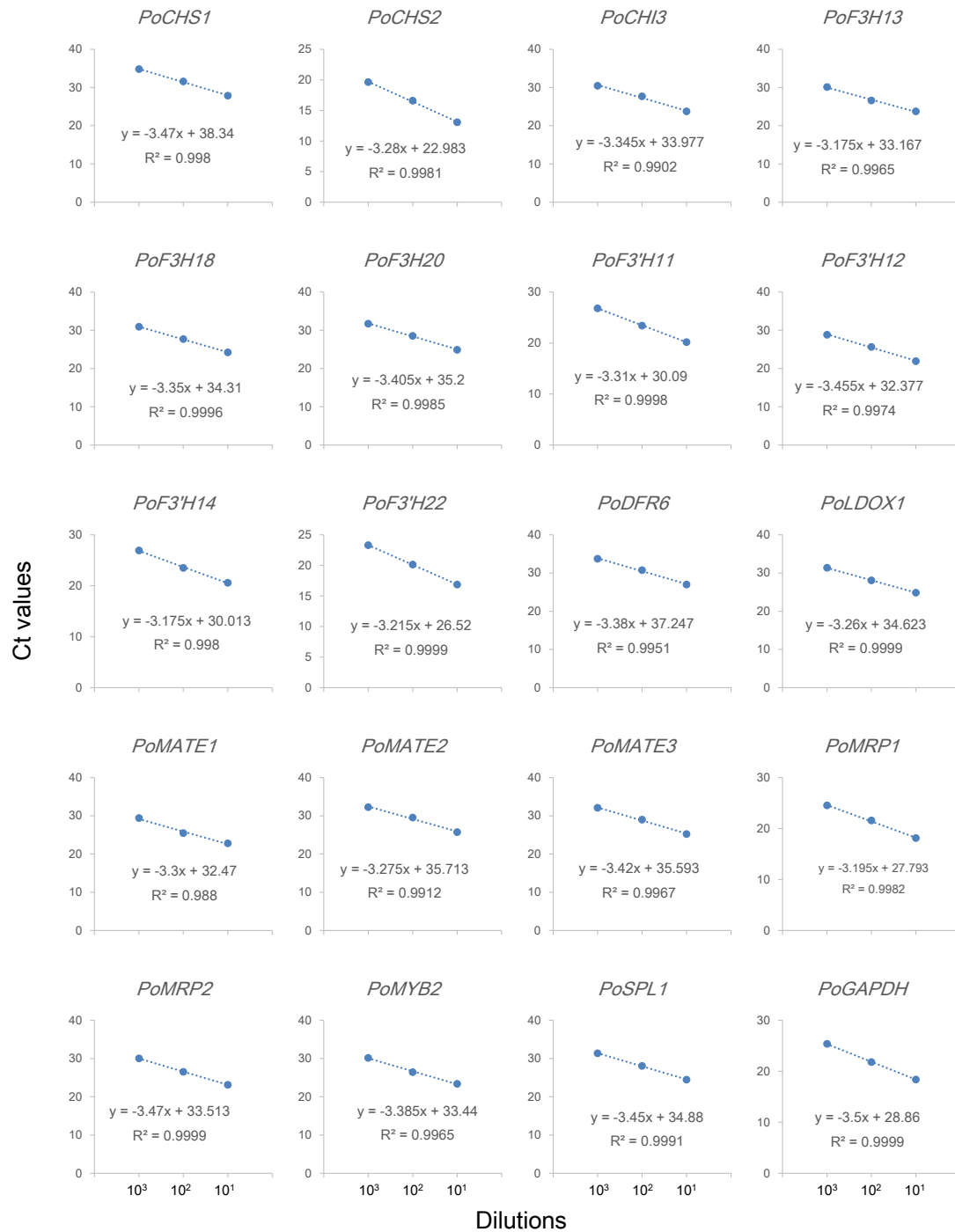

Supplement: Supplementary file 8 [file Image1.PDF]
