# Supplementary material for: Extensive Transcriptome Changes Underlying the Flower Color Intensity Variation in Paeonia ostii
Source: Front Plant Sci. 2016 Jan 6;6:1205. doi: 10.3389/fpls.2015.01205 (PMC4702479; doi:10.3389/fpls.2015.01205)

Supplementary Figure 2. HPLC results of *P. ostii* petals. **(A)** HPLC traces of methanol-extactable anthocyanins. **(B)** HPLC traces of methanol-extactable anthoxanthins. Peaks corresponding to peonidin-3,5-di-*O*-glucoside (Pn3G5G) and cyanidin-3,5-di-*O*-glucoside (Cy3G5G) were identified by comparison to known standards. **(C)** Anthoxanthin concentration. Each value given in the plot is shown as average ± standard deviation. Different superscript letters on the horizontal axis labels indicate statistically significant differences between means of different color classes, as judged by *t* test ($P <$ 0.05). The analyzed plants were shown in Figure 1.

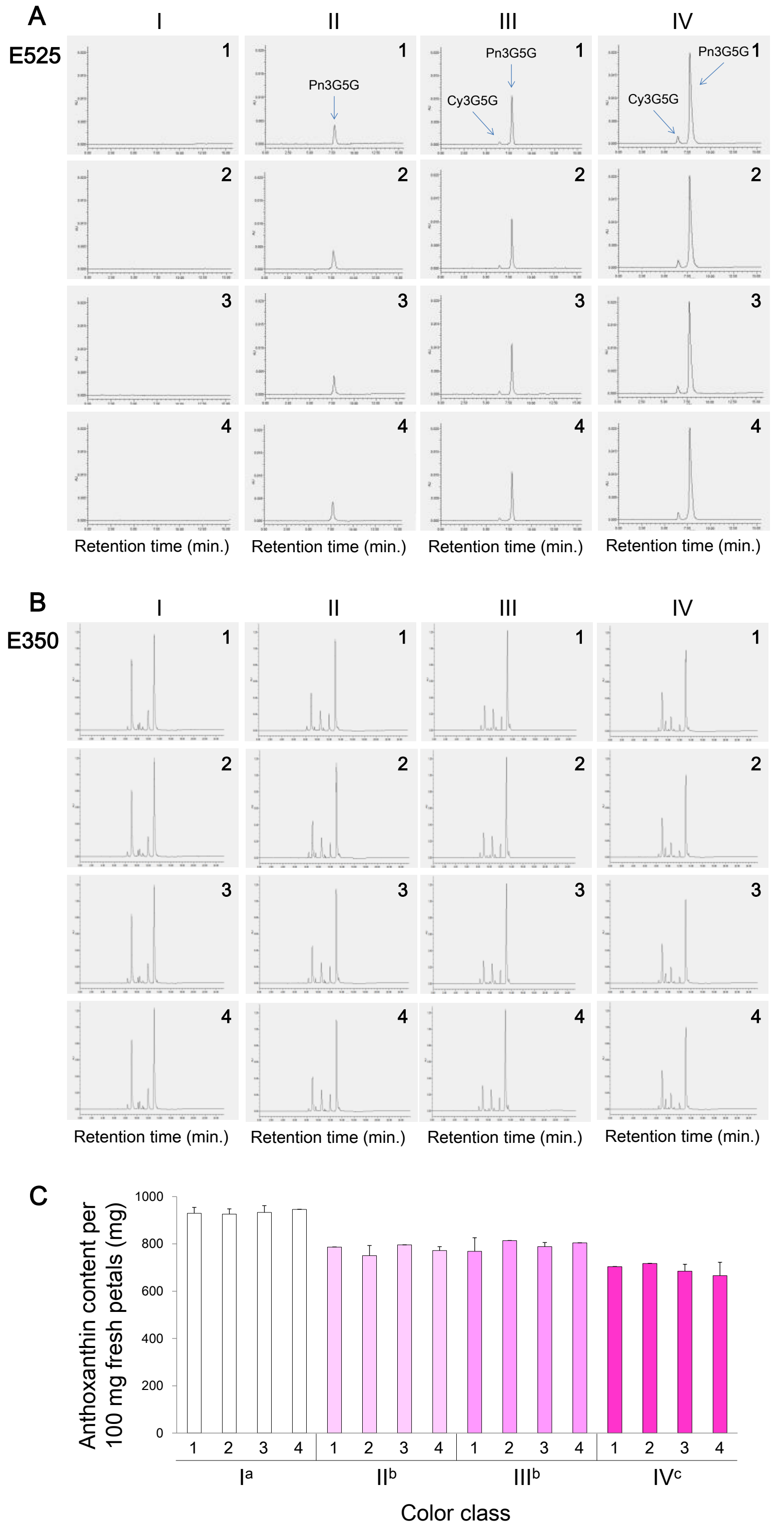

Supplement: Supplementary file 9 [file Image2.PDF]
