# Supplementary material for: Extensive Transcriptome Changes Underlying the Flower Color Intensity Variation in Paeonia ostii
Source: Front Plant Sci. 2016 Jan 6;6:1205. doi: 10.3389/fpls.2015.01205 (PMC4702479; doi:10.3389/fpls.2015.01205)

Supplementary Figure 3. Confirmation of the expression profiles of 19 genes in *P. ostii* by qRT-PCR. **(A)** Gene expression data obtained by qRT-PCR (blue bars) and transcriptome sequencing (red bars). **(B)** Correlation between the expression profiles determined by transcriptome sequencing (y-axis) and qRT-PCR (x-axis). CHS, chalcone synthase; CHI, chalcone isomerase; F3H, flavanone 3-hydroxylase; F3'H, flavonoid 3'-hydroxylase; DFR, dihydroflavonol 4-reductase; LDOX, leucoanthocyanidin dioxygenase; MATE, multidrug and toxin extrusion transporter; MRP, ATP binding cassette transporter; MYB, MYB transcription factor; SPL, SBP-box transcription factor.

A

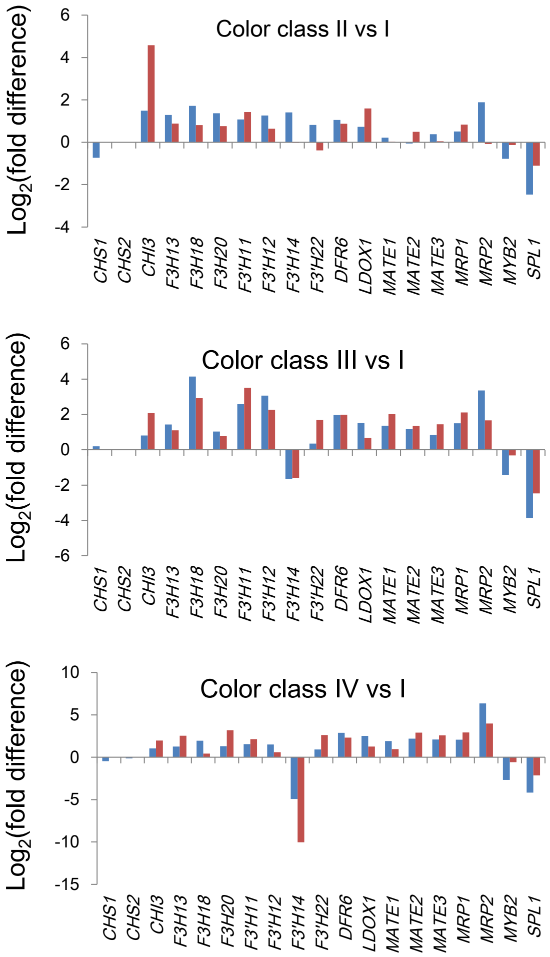

B

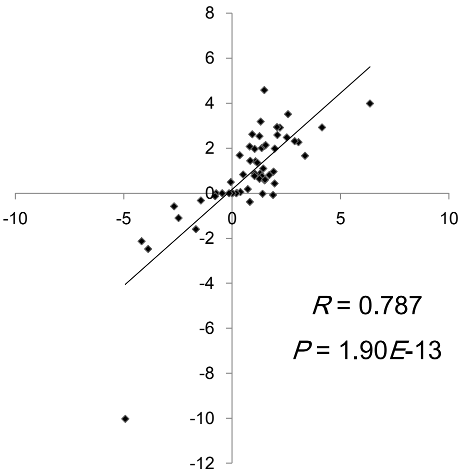

Supplement: Supplementary file 10 [file Image3.PDF]
