# Supplementary material for: Extensive Transcriptome Changes Underlying the Flower Color Intensity Variation in Paeonia ostii
Source: Front Plant Sci. 2016 Jan 6;6:1205. doi: 10.3389/fpls.2015.01205 (PMC4702479; doi:10.3389/fpls.2015.01205)

Supplementary Figure 4. Results of flavonoid biosynthetic pathway mapping of *P. ostii* transcriptome. **(A)** Distribution of *P. ostii* transcripts in the flavonoid biosynthesis pathway, with boxes showing enzyme commission (EC) numbers, and boxes marked with red lines indicating genes covered by our sequence data. **(B)** Distribution of transcripts in the anthocyanin biosynthesis branch of the flavonoid pathway. Each enzyme name is followed in parentheses by the number of unigenes homologous to gene family encoding this enzyme. PAL, phenylalanine ammonia-lyase; C4H, cinnamate 4-hydroxylase; 4CL, 4-coumaroyl:CoA ligase; PKR, 6'-deoxychalcone synthase; CHS, chalcone synthase; CHI, chalcone isomerase; F3H, flavanone 3-hydroxylase; F3’H, flavonoid 3'-hydroxylase; FLS, flavonol synthase; DFR, dihydroflavonol 4-reductase; LDOX, leucoanthocyanidin dioxygenase; UFGT, UDP flavonoid glucosyl transferase; MATE transporter, multidrug and toxin extrusion transporter; ABC transporter, ATP binding cassette transporter.

A

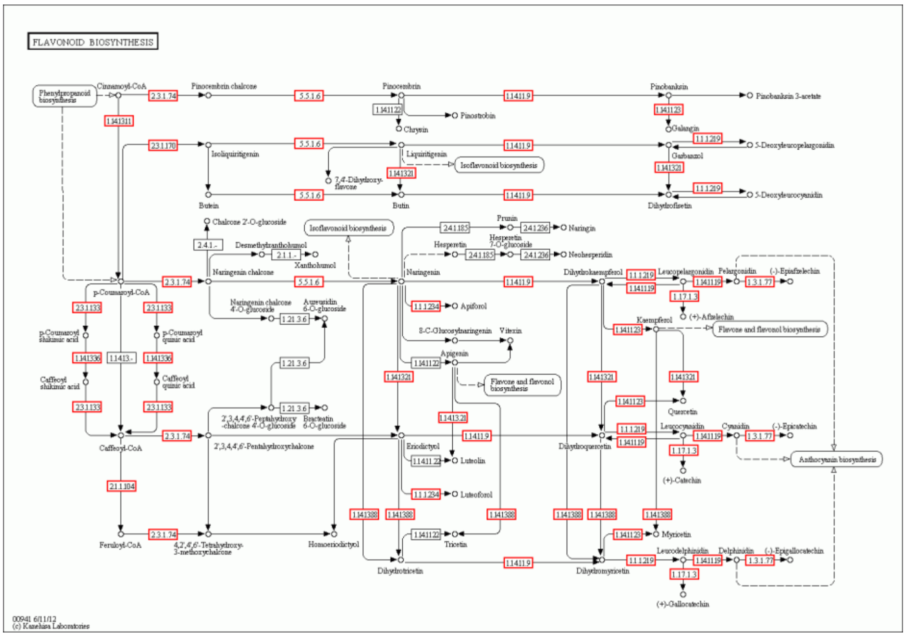

B

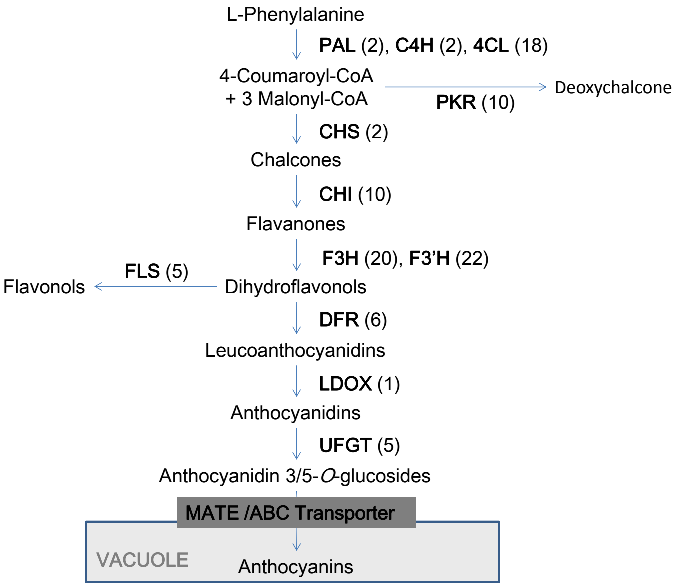

Supplement: Supplementary file 11 [file Image4.PDF]
