# Supplementary material for: Extensive Transcriptome Changes Underlying the Flower Color Intensity Variation in Paeonia ostii
Source: Front Plant Sci. 2016 Jan 6;6:1205. doi: 10.3389/fpls.2015.01205 (PMC4702479; doi:10.3389/fpls.2015.01205)

Supplementary Figure 5. Phylogenetic tree displaying the similarity of PoMYB2 to other R2R3 MYB proteins. The tree was created by neighbor-joining method, based on an alignment of the 102 amino acids spanning the R2 and R3 MYB DNA-binding domain. Proteins origin is indicated by a two-letter prefix: At, *Arabidopsis thaliana*; Am, *Antirrhinum majus*; Eg, *Eucalyptus grandis*; Fa, *Fragaria x ananassa*; Gt, *Gentiana triflora*; Md, *Malus domestica*; Nn, *Nelumbo nucifera*; Os, *Oryza sativa*; Ph, *Petunia x hybrid*; Po, *Paeonia ostii*; Sl, *Solanum lycopersicum*; Tc, *Theobroma cacao*; Vv, *Vitis vinifera*; Zm, *Zea_mays*. The function of some proteins is given in parentheses. Numbers at branch points indicate bootstrap support (1000 replicates). Scale bar corresponds to 5 amino acids substitutions per residue. Gene Bank accession numbers are as follows: AtPAP1, AT1G56650; AtPAP2, AT1G66390; AtMYB113, AT1G66370; AtMYB114, AT1G66380; AtMYB5, AT3G13540; AtMYB28, AT5G61420; AtMYB17, AT3G61250; AtMYB42, AT4G12350; AtMYB6, AT4G09460; AtMYB4, AT4G38620; AtMYB3, AT1G22640; AtMYB7, AT2G16720; AtMYB12, AT2G47460; AtMYB111, AT5G49330; AtGL1, AT3G27920; AtMYB23, AT5G40330; AtWER1, AT5G14750; AtTT2, AT5G35550; AtMIXTA, AT5G15310; AmROSEA1, ABB83826; AmROSEA2, ABB83827; AmMIXTA, CAA55725; EgMYB4L, XP_010044170; FaMYB1, AAK84064; GtMYB3, BAF96933; MdMYB1, ABK58136; MdMYB10, ACQ45201; NnMYB4L, XP_010259378; OsMYB4, BAA23340; PhMYB27, AHX24372; PhAN2, BAP28593; PhPH4, BAP28594; PhMYB1, CAA78386; PhMYB2, CAA78387; PhODORANT1, AAV98200; SlANT1, AAQ55181; TcMYB6, XP_007033078; VvMYBA1, BAD18977; VvMYBA2, BAD18978; VvMYB6, XP_002273328; ZmC1, 1613412E.

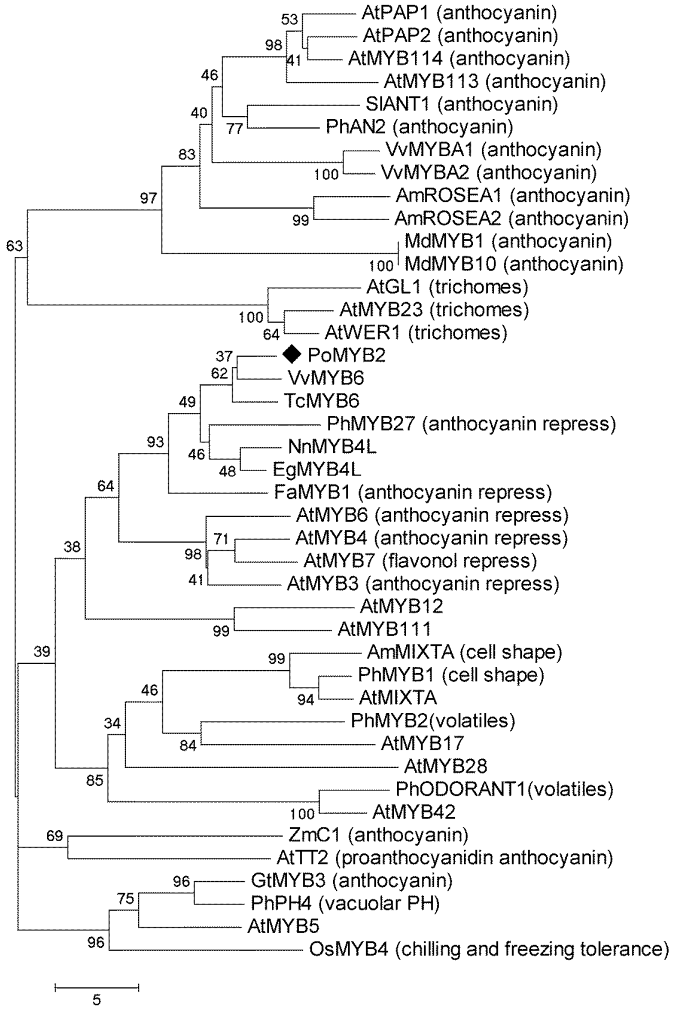

Supplement: Supplementary file 12 [file Image5.PDF]
