# Supplementary material for: Extensive Transcriptome Changes Underlying the Flower Color Intensity Variation in Paeonia ostii
Source: Front Plant Sci. 2016 Jan 6;6:1205. doi: 10.3389/fpls.2015.01205 (PMC4702479; doi:10.3389/fpls.2015.01205)

Supplementary Figure 6. Phylogenetic tree displaying the similarity of PoSPL1 to other SPL proteins. The tree was created by neighbor-joining method, based on amino acid sequences of the SBP-domains. Proteins origin is indicated by a two-letter prefix: At, *Arabidopsis thaliana*; Md, *Malus domestica*; Pm, *Prunus mume*; Po, *Paeonia ostii*; Tc, *Theobroma cacao*; Vv, *Vitis vinifera*. Numbers at branch points indicate bootstrap support (1000 replicates). Scale bar corresponds to 2 amino acids substitutions per residue. Gene Bank accession numbers are as follows: AtSPL1, AT2G47070; AtSPL2, AT5G43270; AtSPL3, AT2G33810; AtSPL4, AT1G53160; AtSPL5, AT3G15270; AtSPL6, AT1G69170; AtSPL7, AT5G18830; AtSPL8, AT1G02065; AtSPL9, AT2G42200; AtSPL10, AT1G27370; AtSPL11, AT1G27360; AtSPL12, AT3G60030; AtSPL13, AT5G50670; AtSPL14, AT1G20980; AtSPL15, AT3G57920; AtSPL16, AT1G76580; AtSPL17, AT5G50570; PmSPL13A, XP_008239496; TcSPL1, XP_007038017; VvSPL13A, XP_002274360.

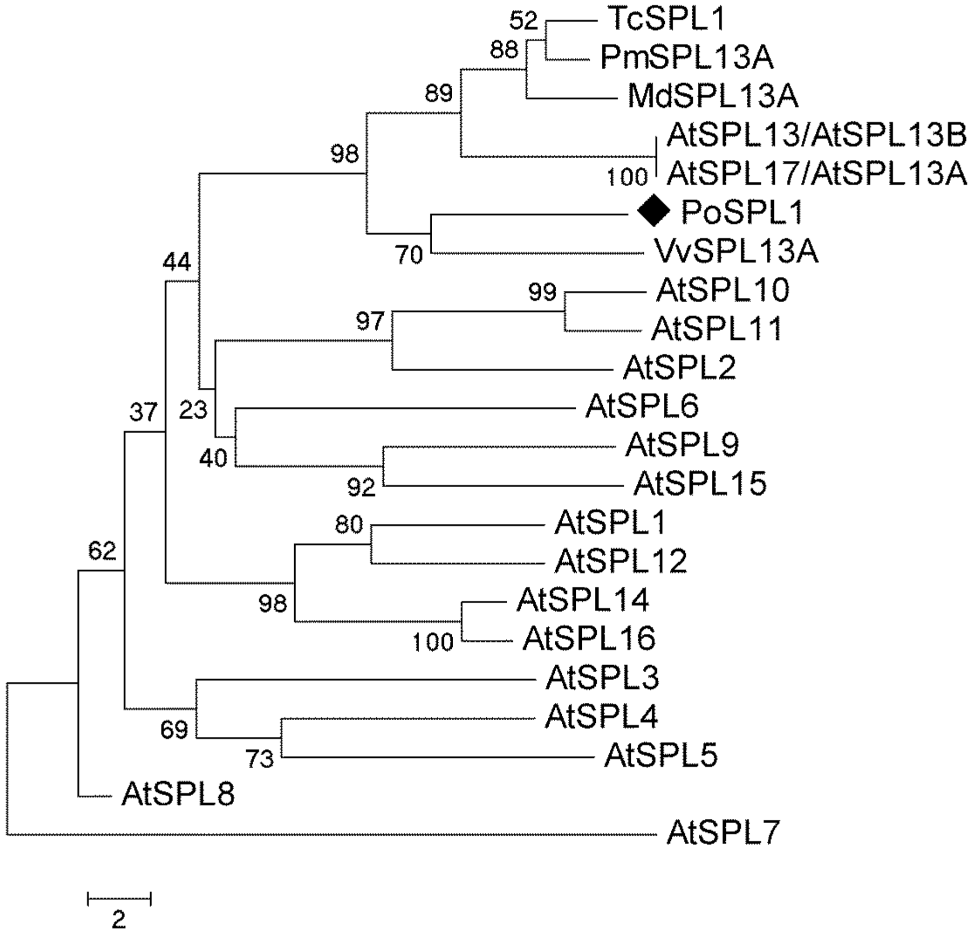

Supplement: Supplementary file 13 [file Image6.PDF]
